# Successful Treatment of Confirmed *Naegleria fowleri* Primary Amebic Meningoencephalitis

**DOI:** 10.3201/eid3004.230979

**Published:** 2024-04

**Authors:** Ahmed Mujadid Khan Burki, Luqman Satti, Saira Mahboob, Syed Onaiz Zulfiqar Anwar, Mahwash Bizanjo, Muhammad Rafique, Najia Karim Ghanchi

**Affiliations:** PNS Shifa Hospital, Karachi, Pakistan (A.M.K. Burki, L. Satti, S. Mahboob, S.O.Z. Anwar, M. Bizanjo, M. Rafique);; Aga Khan University, Karachi (N.K. Ghanchi)

**Keywords:** *Naegleria fowleri*, Pakistan, meningitis/encephalitis, parasites, primary amebic meningoencephalitis

## Abstract

Primary amebic meningoencephalitis caused by *Naegleria fowleri* is a rare but nearly always fatal parasitic infection of the brain. Globally, few survivors have been reported, and the disease has no specific treatment. We report a confirmed case in Pakistan in a 22-year-old man who survived after aggressive therapy.

*Naegleria fowleri* amebae are thermophilic, free-living, and found in soil and fresh water, such as lakes, rivers, ponds, and untreated swimming pools. The ameba enters the brain through the nose and cribriform plate, causing primary amebic meningoencephalitis. Globally, 381 confirmed cases and only 7 (1.8%) known survivors were reported through 2018 (*1*). The Centers for Disease Control and Prevention recommends microscopic examination of fresh, unfrozen, nonrefrigerated cerebrospinal fluid (CSF) for presumptive diagnosis (*2*). If amebae are identified, the diagnosis should be confirmed through PCR. Although no specific treatment for primary amebic meningoencephalitis exists, the Centers for Disease Control and Prevention recommends combination therapy, including intravenous and intrathecal amphotericin B, azithromycin, miltefosine, rifampin, and dexamethasone (*2*). 

*N. fowleri* ameba poses a substantial problem in Karachi, Pakistan, because of the city’s hot, humid climate in the summer and its coastal location. The first case of *N. fowleri* infection in Pakistan was reported in 2008, and 146 cases were reported by October 2019 (*3*). Other than the United States (41.0%), Pakistan has reported the most *N. fowleri* infections (38.8%) (*2*).

A 22-year-old man sought care at a secondary-care hospital in Karachi on June 17, 2023, with initial symptoms of fever, drowsiness, and vomiting. He had no history of recreational water sports or swimming. His pulse rate was 105 beats/min, temperature 38.8°C, and blood pressure 121/78 mm Hg. His oxygen saturation was 95% in room air, and he had no respiratory distress. His Glasgow coma score was 11/15; he had neck rigidity, bilateral downgoing planters, and tonic-clonic seizures. Results of laboratory testing were unremarkable except an elevated leukocyte count, 19.3 × 10^9^ cells/mL (reference range 4–10 × 10^9^ cells/mL). We made a provisional diagnosis of acute meningoencephalitis and began empirical therapy with intravenous meropenem (2 g/12 h), intravenous vancomycin (1 g/12 h), intravenous dexamethasone (4 mg/8 h), and sodium valproate (500 mg/12 h).

On the same day, we transferred the patient to the intensive care unit of a tertiary-care hospital in Karachi for additional testing and critical care. We sent a CSF sample for microscopic examination, chemical testing, and bacterial culture. The CSF sample was slightly turbid, and test results showed high levels of protein, 950 mg/dL (reference 15–40 mg/dL); glucose, 79.2 mg/dL (reference 50–80 mg/dL); erythrocytes, 52 cells/mm^3^ (reference 0 cells/mm^3^); and leukocytes, 162 cells/mm^3^ (reference 0–5 cells/mm^3^), with 60% segmented neutrophils. A wet mount of the CSF showed trophozoite forms of an ameba (Figure). We changed the patient’s treatment regimen to oral miltefosine (50 mg/6 h), intravenous amphotericin B (75 mg immediately, then 50 mg/24 h), oral rifampin (400 mg/12 h), intravenous fluconazole (400 mg/12 h), intravenous azithromycin (500 mg/24 h), intravenous sodium valproate (500 mg/8 h), and intravenous 20% mannitol (200 mL/8 h). The patient’s condition began to deteriorate, he had onset of seizures, and his Glasgow coma score dropped to 8/15. We placed him on mechanical ventilation 4 hours after transfer to intensive care.

**Figure:**
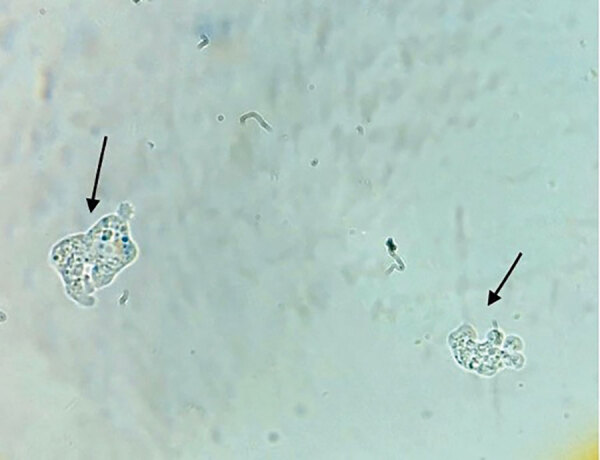
Two trophozoites with pseudopod formation identified during microscopic examination of cerebrospinal fluid from a 22-year-old man later diagnosed with *Naegleria fowleri* infection, Karachi, Pakistan, 2023. Original magnification ×40.

We sent the CSF sample to the Aga Khan University Reference Laboratory in Karachi for PCR testing. The sample was spun down, and the cell pellet was used for DNA extraction by QIAamp DNA Extraction Kit (QIAGEN, https://www.qiagen.com), according to manufacturer protocol. Real-time PCR was performed using primers targeting the 18S rRNA gene of *Naegleria* sp., as described previously (*4*). Parallel PCRs for human RNase P gene, as assay control, and ATCC *N. fowleri* HB1 (30174D), as positive control, were performed. The PCR testing confirmed the pathogen as *N. fowleri* ameba.

On day 3, we began intrathecal amphotericin B (15 mg). The intrathecal catheter was accidently removed during nursing care, and we made the decision to discontinue the intrathecal amphotericin B. The clinical course was complicated by ventilator-associated *Acinetobacter baumannii* pneumonia that was successfully treated with intravenous and inhalational colistin. With combination therapy, the patient’s condition began to improve, and on day 8, he was successfully weaned off mechanical ventilation. He completed a 3-week course of therapy, and on day 28, he was discharged. The patient has since returned to his previous state of health without any neurologic deficit.

A total of 146 cases of *N. fowleri* amoeba infections were reported in Pakistan during 2008–2019, and only 2 (1.36%) were in patients who had a history of recreational water activity (*3*). In the patient we describe, the most likely transmission could be ritual ablution with tap water, given that *N. fowleri* amebae have been isolated in the local domestic water supply (*4*). Our patient is 1 of only 8 reported laboratory-confirmed *N. fowleri* survivors worldwide (Table). The survival of our patient could be multifactorial: first, a high index of suspicion led to an early diagnosis, within 24 hours from seeking care to ICU admission; second, we used a combination of antimicrobial drugs, including miltefosine, amphotericin B, rifampin, and azithromycin, administered within 2 hours of diagnosis and ≈48 hours after onset of symptoms. 

**Table:** Demographic profiles, time from symptom onset to diagnosis, and management of 8 confirmed survivors of *Naegleria fowleri* infection, 1971–2023*

Country, year of infection (reference)	Age, y/sex	Time from symptom onset to diagnosis	Therapy given	Adjuvant therapy
Australia, 1971 (*5*)	14/M	Unknown	Unknown	Unknown
United States, 1978 (*6*)	9/M	3 d	Intravenous and intrathecal amphotericin b, intravenous and intrathecal miconazole, oral rifampin, intravenous sulfisoxazole	Intravenous dexamethasone, intravenous phenytoin
Mexico, 2003 (*7*)	10/M	9 h	Intravenous amphotericin, intravenous fluconazole, intravenous dexamethasone, oral rifampin	ETT, intravenous dexamethasone
United States, 2013 (*8*)	12/F	2 d	Intravenous amphotericin, intravenous fluconazole, oral rifampin, intravenous azithromycin, oral miltefosine after 3 d, intrathecal amphotericin on second day for 10 d	Intravenous dexamethasone, extraventricular drain, intravenous 20% mannitol with hypertonic saline, hypothermia
United States, 2013 (*9*)	8/M	5 d	Intravenous amphotericin, oral rifampin, intravenous fluconazole, intravenous azithromycin, oral miltefosine	ETT, EVD, dexamethasone, mannitol
Pakistan, 2015 (*10*)	25/M	3 d	Intravenous amphotericin, oral rifampin, intravenous fluconazole	Intravenous chlorpromazine
United States, 2016 (NA)	16/M	1 d	Intravenous amphotericin, intravenous fluconazole, oral rifampin, intravenous azithromycin, oral miltefosine after 3 d, intrathecal amphotericin on second day for 10 d	Mechanical ventilation, hypothermia
Pakistan, 2023 (this case)	22/M	2 d	Intravenous amphotericin, intravenous fluconazole, oral rifampin, intravenous azithromycin, oral miltefosine, intrathecal amphotericin for 2 d	Mechanical ventilation, intravenous sodium valproate, intravenous 20% mannitol

*ETT, endotracheal tube intubation; EVD, external ventricular drain; NA, not applicable (only news reports).

In conclusion, primary amebic meningoencephalitis caused by *N. fowleri* amoeba is a rare but fatal disease. A high index of suspicion, early diagnosis, and aggressive combination therapy can help prevent death and long-term illness.
